# Fruquintinib in combination with tislelizumab versus trifluridine/tipiracil and bevacizumab in third-line and beyond MSS mCRC without active liver metastases—the IKF-080/AIO-QUINTIS trial

**DOI:** 10.1016/j.esmogo.2026.100312

**Published:** 2026-03-10

**Authors:** J. Tintelnot, J. Gorgulho, S.-E. Al-Batran, D. Arnold, A. Reinacher-Schick, S. Kasper, G. Prager, T. Goetze, R. Boston, M.S. Cruz, F. Dierks, A. Stein

**Affiliations:** 1 ll. Department of Medicine, University Medical Center Hamburg-Eppendorf, Hamburg, Germany; 2 The Frankfurt Institute of Clinical Cancer Research (IKF) and Krankenhaus Nordwest, UCT-University Cancer Center, Frankfurt, Germany; 3 Asklepios Tumorzentrum Hamburg, Department of Oncology and Hematology, AK Altona, Hamburg, Germany; 4 St. Josef-Hospital, Department of Hematology and Oncology with Palliative Care, Ruhr University Bochum, Bochum, Germany; 5 West German Cancer Center, Department of Medical Oncology, University Hospital Essen, Essen, Germany; 6 Division of Oncology, Department of Medicine I, Medical University of Vienna, Vienna, Austria; 7 Hematology-Oncology Practice Eppendorf (HOPE), University Cancer Center Hamburg (UCCH), University Medical Center Hamburg-Eppendorf, Hamburg, Germany

**Keywords:** MSS mCRC, non-liver-metastases, checkpoint inhibition, fruquintinib, tislelizumab

## Abstract

**Background:**

Patients with metastatic colorectal cancer (mCRC) who have progressed on fluoropyrimidines, oxaliplatin, irinotecan, anti-angiogenic agents, and anti-epidermal growth factor receptor (EGFR) therapies have limited treatment options and poor prognosis, with a median overall survival (mOS) of ∼6 months on single-agent regorafenib or trifluridine/tipiracil. The addition of bevacizumab to trifluridine/tipiracil improved mOS to 10.8 months, and fruquintinib, a selective vascular endothelial growth factor receptor (VEGFR) 1-3 inhibitor, improved mOS to 7.4 months versus 4.8 months with placebo in refractory mCRC. However, combinations of tyrosine kinase inhibitors and immune checkpoint inhibitors have shown benefit primarily in patients without liver metastases in microsatellite stable mCRC, likely due to liver-associated immunosuppression. The QUINTIS trial evaluates whether fruquintinib plus tislelizumab can improve outcomes to the standard of care with trifluridine/tipiracil and bevacizumab in third-line and beyond mCRC.

**Methods/design:**

QUINTIS is a prospective, randomized, open-label, multicenter, phase II trial enrolling patients with advanced or metastatic colorectal adenocarcinoma without active liver metastases who have been previously treated with fluoropyrimidines, oxaliplatin, irinotecan, bevacizumab, and, if indicated, an EGFR inhibitor. Participants are randomly assigned 1 : 1 to one of the following treatment arms: arm A (experimental): fruquintinib 5 mg orally once daily on days 1-21 of a 4-week cycle (q4w) plus tislelizumab 400 mg intravenously on day 1 every 6 weeks (q6w); or arm B (control): trifluridine/tipiracil 35 mg/m^2^ orally twice daily on days 1-5 and 8-12 of a 4-week cycle (q4w) plus bevacizumab 5 mg/kg intravenously on day 1 every 2 weeks (q2w). Randomization is stratified by prior anti-angiogenic therapy (<12 versus ≥12 months ago), *BRAF*/*RAS* mutation status, and history of liver metastases (never versus treated). Tumor assessments occur every 8 weeks; follow-up continues for up to 18 months after enrolment. Optional translational research includes tumor, blood, and stool sampling to explore biomarkers of response and resistance.

## Graphical abstract

**Figure:**
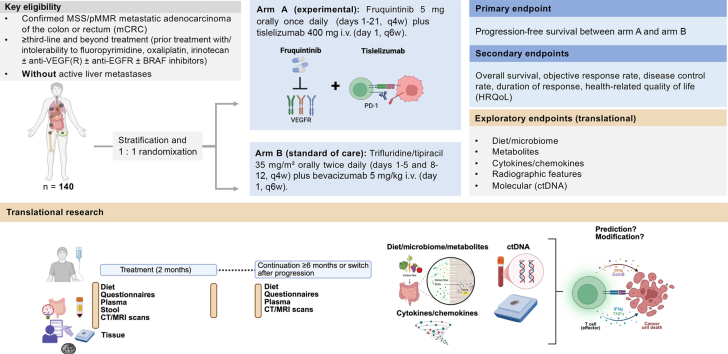

## Key questions addressed by the QUINTIS trial

• Does combined VEGFR and PD-1 blockade improve outcomes over current standard therapy in refractory mCRC without active liver metastases?
• Is guided patient selection based on the absence of active liver metastases clinically feasible?
• Which subgroups benefit most from the combination of VEGFR and PD-1 blockade?

## Description of Protocol

### Background

Patients with metastatic colorectal cancer (mCRC) who have progressed on or after, or are intolerant to, fluoropyrimidines, oxaliplatin, irinotecan, anti-angiogenic agents, and anti-epidermal growth factor receptor (EGFR) therapies have limited therapeutic options and a poor prognosis, with a median overall survival (mOS) of ∼6 months on single-agent regorafenib or trifluridine/tipiracil.^1^^,^^2^ The addition of bevacizumab to trifluridine/tipiracil improved mOS to 10.8 months compared with trifluridine/tipiracil alone [hazard ratio (HR) 0.61, 95% confidence interval (CI) 0.49-0.77, *P* < 0.001].^3^ In addition, fruquintinib, a highly selective and potent oral inhibitor of vascular endothelial growth factor receptors (VEGFR) 1, 2, and 3, significantly improved mOS (7.4 versus 4.8 months, *P* < 0.001) compared with placebo in refractory mCRC after a median of four prior lines of treatment.^4^ Despite these advances, effective new treatment options are still needed for this heavily pretreated population.

### Rationale for the combination of fruquintinib and tislelizumab in mCRC without active liver metastases

Combination regimens including programmed cell death protein 1 (PD-1)/programmed death-ligand 1 and/or cytotoxic T lymphocyte-associated antigen 4 inhibitors with or without tyrosine kinase inhibitors (TKIs) or anti-angiogenic agents have not demonstrated significant benefit in unselected microsatellite stable (MSS) mCRC populations, as shown in several trials—such as LEAP-017 (pembrolizumab + lenvatinib), IMblaze 370 (atezolizumab ± cobimetinib versus regorafenib), CCTG CO.26 (durvalumab + tremelimumab), BACCI (capecitabine + bevacizumab ± atezolizumab), and KEYFORM-007 (pembrolizumab + favezelimab).^5^, ^6^, ^7^, ^8^, ^9^, ^10^ However, both preclinical and clinical analyses indicate that the presence of liver metastases negatively predicts response to immunotherapy-based combinations. In multiple trials (Table 1), the non-liver-metastatic subgroups showed numerically greater benefit, though patient numbers were limited. The recently reported phase III STELLAR-303 trial (zanzalintinib + atezolizumab versus regorafenib) demonstrated the first statistically significant improvement in OS (10.9 versus 9.4 months; HR 0.80, 95% CI 0.69-0.93) in a non-microsatellite instability-selected refractory mCRC population.^11^ Notably, OS was numerically longer in patients without liver metastases (mOS 15.9 versus 12.7 months; HR 0.79, 95% CI 0.61-1.03), although this difference has not yet reached statistical significance, reflecting the generally better prognosis and therefore not yet mature follow-up in this subgroup. The final data have to be awaited before final conclusions can be made regarding the role of non-liver metastases in STELLAR 303. To finally clarify the hypothesis that hepatic metastases confer resistance to immunotherapy, dedicated studies in non-liver-metastatic patients are needed. Mechanistically, liver metastases can sequester and delete tumor-reactive T cells via hepatic macrophage-mediated immune tolerance, thereby impairing systemic antitumor immunity.^12^ These findings provide a strong biological rationale to test immunotherapy combinations specifically in mCRC patients without liver involvement.

**Table 1:** Selected phase II-III trials in advanced MSS or non-MSI-selected mCRC

Study	Treatment	*n*	Non-liver-metastatic (*n*)	OS overall (median/HR)	OS without LM (median/HR)	OS in LM (median/HR)
LEAP 017^8^	Pembrolizumab + lenvatinib versus regorafenib/trifluridine-tipiracil	480	144	9.8 versus 9.3 months HR 0.83 (0.68-1.02)	**0.65 (0.42-0.99)**	0.91 (0.72-1.15)
BACCI^10^	Capecitabine + bevacizumab ± atezolizumab	133	23	10.3 versus 10.2 months HR 0.96 (0.63-1.45)	0.33 (0.11-1.02)	1.14 (0.72-1.81)
STELLAR 101^5^	Zanzalintinib + atezolizumab versus zanzalintinib	107	34	11.7 versus 11.1 months HR 0.89 (0.56-1.42)	0.74 (0.27-2.04)	NA
STELLAR 303^11^	Zanzalintinib + atezolizumab versus regorafenib	901	383	**10.9 versus 9.4 months** **HR0.80 (0.69-0.93)**	15.9 versus 12.7 months HR 0.79 (0.61-1.03)	**8.9 versus 7.7 months** **HR 0.78 (0.65-0.94)**

Significant differences are bold.

CI, confidence interval; HR, hazard ratio; LM, liver metastases; mCRC, metastatic colorectal cancer; MSI, microsatellite instability; MSS, microsatellite stable; OS, overall survival.

**Table 2:** Specifications

Subject area	Medicine and Dentistry
More specific subject area	Third-line and beyond therapy of mCRC
Name of your trial in progress	QUINTIS trial
Reagents/tools	Patients in the experimental arm will receive fruquintinib 5 mg orally once daily on days 1-21 of each 28-day cycle (q4w), in combination with tislelizumab 400 mg intravenously on day 1 of each 42-day cycle (q6w) (Figure 1). Patients in the control arm will receive trifluridine/tipiracil 35 mg/m^2^ orally twice daily on days 1-5 and 8-12 of each 28-day cycle (q4w), in combination with bevacizumab 5 mg/kg intravenously on day 1 of each 14-day cycle (q2w).
Trial design	Randomized controlled phase ll trial
Trial registration	2024-519929-38-00; NCT06856837
Ethics	This clinical trial has been designed and is being conducted and reported in accordance with the study protocol, the International Council for Harmonisation Harmonized Tripartite Guideline for Good Clinical Practice, applicable local regulatory requirements for the sponsor [including Regulation (EU) No. 536/2014], and the ethical principles outlined in the current version of the Declaration of Helsinki. The trial is registered in the EU Clinical Trials Information System (CTIS) and has obtained authorization and ethical approval in compliance with Regulation (EU) No. 536/2014. The trial commenced in November 2025.
Value of the Trial in Progress	This trial assesses whether combined VEGFR and PD-1 blockade improves outcomes compared with current standard therapy in refractory metastatic colorectal cancer (mCRC) without active liver metastases. It also evaluates the feasibility of selecting patients for third-line and beyond treatment based on the absence of active liver metastases.

PD-1, programmed cell death protein 1; VEGFR, vascular endothelial growth factor receptor.

### Safety considerations and prior data for fruquintinib and PD-1 blockade

The safety of combining PD-1 inhibitors with TKIs is supported by prior studies such as LEAP-017, where pembrolizumab and lenvatinib demonstrated manageable toxicity. Grade ≥3 adverse events were more common with the combination (77% versus 59%), driven mainly by hypertension (28% versus 9%), proteinuria (11% versus 1%), and diarrhea (9% versus 4%). Immune-related events were also more frequent (48% versus 10%), notably hypothyroidism (38% versus 7%) and hyperthyroidism (5% versus 1%), though grade ≥3 immune events were infrequent (8% versus 2%).^8^ After adjustment for treatment duration, exposure-adjusted rates of severe events were comparable between arms. The combination of fruquintinib and PD-1 inhibitors has shown feasibility and activity in several small retrospective and real-world studies^13^^,^^14^ and in two single-arm phase II trials combining fruquintinib + tislelizumab with radiotherapy (*n* = 24) or fecal microbiota transplantation (*n* = 20).^15^^,^^16^ Across these studies, treatment-related adverse events occurred in >90% of patients, which were mostly grade 1-2, and grade ≥3 toxicities were reported in ≤30%. A recent meta-analysis including 716 patients treated with fruquintinib ± PD-1 inhibitors across nine retrospective cohorts and one randomized trial confirmed that the safety profile of the combination was consistent with expected anti-VEGFR and PD-1-related toxicities, without unexpected signals.^17^ Efficacy outcomes favored the combination, with higher objective response rates (ORR 2.45, 95% CI 1.83-3.56) and improved progression-free survival (PFS) (HR 0.64, 95% CI 0.49-0.84) compared with fruquintinib monotherapy. The presence of liver metastases remained a strong independent negative prognostic factor for both OS and PFS.^17^

Collectively, these data provide a strong biological and clinical rationale to evaluate the efficacy and safety of fruquintinib and tislelizumab compared with trifluridine/tipiracil and bevacizumab in patients with refractory mCRC without active liver metastases, aiming to improve OS in this biologically defined patient population.

### Study objectives

The primary objective of this trial is to evaluate the efficacy of fruquintinib in combination with the PD-1 inhibitor tislelizumab in patients with mCRC (EudraCT: 2024-519929-38-00). The secondary objectives are to further assess the efficacy of the combination regimen, including additional measures of clinical benefit, and to evaluate patient-reported health-related quality of life (HRQoL) during treatment. The safety objectives are to assess the safety profile and tolerability of fruquintinib combined with tislelizumab in this patient population. The exploratory (translational) objectives are to correlate molecular, serum, and stool biomarkers with radiographic and clinical outcomes, with the goal of identifying molecular markers predictive of tumor response and survival.

## Methods/design

The QUINTS trial is a prospective, randomized, open-label, multicenter, multinational phase II study conducted across 30 sites in Germany and Austria (Table 2, Supplementary Table S1, available at https://doi.org/10.1016/j.esmorw.2026.100688). Eligible patients must have histologically confirmed advanced or metastatic adenocarcinoma of the colon or rectum and must have received prior treatment including fluoropyrimidines (5-fluorouracil), oxaliplatin, irinotecan, and bevacizumab, as well as EGFR inhibitors if indicated.

Participants meeting all eligibility criteria (Table 3) will be randomly assigned in a 1 : 1 ratio to arm A or arm B, stratified by (i) the time from anti-angiogenic therapy (<12 months versus ≥12 months), (ii) *BRAF*/*RAS* mutation status (wild type versus mutant), and (iii) history of liver metastases (never versus prior but treated).

**Table 3:** Inclusion and exclusion criteria for the QUINTIS study

Inclusion criteria 1. Patient provided signed informed consent form. 2. Patient is ≥18 years of age at the time of given informed consent. 3. Patient has been diagnosed with histologically or cytologically proven microsatellite stable (MSS)/mismatch repair-proficient (pMMR) metastatic adenocarcinoma of the colon or rectum, which is not amenable to potentially curative resection. 4. Known *RAS* (*KRAS* or *NRAS*) and *BRAF V600E* mutational status. Note: These mutations are mutually exclusive. Therefore, if one of the factors is mutated, it is not required to determine the mutation status of the others, as they are then assumed to be wild type. 5. Patient without liver metastases defined as subjects without active liver metastases at screening as determined on baseline imaging of the liver as carried out by CT scan with contrast or MRI. Definitively treated liver metastases (which includes surgical resection, microwave or radiofrequency ablation, or stereotactic body radiotherapy, but not yttrium-90 or chemoembolization alone) that were treated at least 3 months before enrolment with no evidence of radiologic progression on subsequent imaging are considered to be non-active liver metastases. 6. Patient received at least one line of previous treatment with a fluoropyrimidine, oxaliplatin, irinotecan, VEGF(R) and if indicated EGFR and/or BRAF inhibitors in the advanced setting, or the patient has been intolerable or ineligible to those treatments. 7. Patient has an ECOG performance status ≤1. 8. Patient has a life expectancy >16 weeks. 9. Patient has adequate hematological, hepatic, and renal function. a. Absolute number of neutrophils (ANC) ≥1.5 × 10^9^/l. b. Platelets ≥100 × 10^9^/l. c. Total bilirubin ≤1.5 times the upper limit of normal (ULN) (or <2 × ULN in case of prior liver involvement or Gilbert disease). d. AST and ALT ≤2.5 × ULN, AP ≤5 × ULN. e. Serum creatinine ≤1.5 × ULN or creatinine clearance (measured by 24 h urine) ≥30 ml/min (i.e. if serum creatinine level is >1.5 × ULN, then a 24-h urine test must be carried out to check the creatinine clearance to be determined). f. Urinary protein ≤2+ on dipstick or routine urinalysis (UA; if urine dipstick or routine analysis is ≥3+, a 24-h urine collection for protein must demonstrate <2000 mg of protein in 24 h to allow participation in this protocol). 10. Adequate coagulation function as defined by international normalized ratio (INR) ≤1.5, and a partial thromboplastin time (PTT) ≤5 s above the ULN (unless receiving anticoagulation therapy). 11. Patients of childbearing potential must agree to remain abstinent (abstain from heterosexual intercourse) or use a very reliable method of contraception (methods with a failure rate of <1%) during treatment and for up to 6 months after treatment ends. Patients using hormonal contraception must be willing to use an additional barrier method (actual adherence only required if randomly assigned to arm B). Non-sterilized male patients with a partner of childbearing potential must be willing to remain abstinent or use reliable methods of contraception during treatment and for up to 6 months after treatment ends (actual adherence only required if randomly assigned to arm B). Male patients with a pregnant partner must be willing to remain abstinent or use condoms for the duration of the pregnancy (actual adherence only required if randomly assigned to arm B). Female patients of childbearing potential must have a negative pregnancy test within the last 7 days before the start of trial therapy. 12. Patient is willing and able to comply with the protocol (including contraceptive measures) for the duration of the study including undergoing treatment and scheduled visits and examinations including follow-up.
Exclusion criteria 1. Patient has known allergic/hypersensitive reactions to at least one of the treatment components. 2. Patient had previous malignancy other than that under study within 3 years or concomitant malignancy, except: those with a 5-year overall survival rate of >90%, e.g. non-melanomatous skin cancer or adequately treated *in situ* cervical cancer. 3. Patient received previous treatment with fruquintinib, trifluridine/tipiracil, regorafenib, or an anti-PD-(L)1 antibody. 4. Patient receives current treatment with any anticancer therapy, such as systemic immunotherapy, chemotherapy, or hormone therapy within ≤2 weeks before study treatment start. 5. Patient receives simultaneous treatment with a different anticancer therapy other than that provided for in the trial (excluding palliative radiotherapy for symptom control). 6. Patient has known untreated or symptomatic CNS or leptomeningeal metastases. 7. Patient has impaired cardiac function or clinically significant cardiac disease including unstable angina within 6 months before the first dose of study treatment, acute myocardial infarction <6 months before the first dose of study treatment, New York Heart Association (NYHA) class II-IV congestive heart failure, uncontrolled hypertension (defined as an average systolic blood pressure >160 mmHg or diastolic blood pressure >100 mmHg) despite optimal treatment, uncontrolled cardiac arrhythmias requiring antiarrhythmic therapy other than β-blockers or digoxin, active coronary artery disease, or corrected QT interval (QTc) ≥470. 8. Patient has a history of uncontrolled infection with human deficiency virus (HIV) or chronic infection with hepatitis B or C virus (HBV, HCV). 9. Patient has evidence of bleeding diathesis. 10. Patient has a history of gastrointestinal perforation or fistulae in the past 6 months or risk factors for perforation. 11. Patient has grade 3-4 gastrointestinal bleeding within 3 months before the first dose of trial therapy. 12. Use of strong inducers or inhibitors of CYP3A4 within 2 weeks (or 5 half-lives, whichever is longer) before the first dose of study drug. 13. Patient had a major surgery within 2 weeks before the first dose of trial therapy. 14. Patient experienced severe, life-threatening, or recurrent (grade ≥2) immune-mediated adverse events (AEs) or infusion-related reactions including those that led to permanent discontinuation while on treatment with immune-oncology agents. 15. Patient received prior immunosuppressive therapy: immunosuppressive doses of systemic medications of >10 mg/day of prednisone or equivalent must be discontinued ≥2 weeks before the first dose of study treatment. Short courses of high-dose corticosteroids and/or continuous low dose of prednisone (<10 mg/day) are permitted. In addition, inhaled, intranasal, intraocular, and/or joint injections of corticosteroids are allowed. 16. Patient has active autoimmune disease that has required systemic treatment (except replacement therapy) within the past 2 years or any other diseases requiring immunosuppressive therapy while on study. 17. Patient has a history of solid organ transplantation. 18. Patient has a history of thromboembolic events (including deep vein thrombosis and pulmonary embolism) within the past 6 months or history of stroke and/or transient ischemic attack within the last 12 months. 19. Patient has a history of or current interstitial pneumonitis. 20. Patient has evidence of any other serious concomitant or medical condition that, in the opinion of the investigator, presents a high risk of complications to the patient or reduces the likelihood of clinical effect. 21. Female patient is pregnant or breast feeding or planning to become pregnant within and up to 6 months after end of treatment.

ALT, alanine aminotransferase; AP, alkaline phosphatase; AST, aspartate aminotransferase; CNS, central nervous system; CT, computed tomography; ECOG, Eastern Cooperative Oncology Group; EGFR, epidermal growth factor receptor; MRI, magnetic resonance imaging; PD-(L)1, programmed death-(ligand) 1; VEGF(R), vascular endothelial growth factor (receptor).

### Treatment

#### Arm A (experimental arm)

**Figure 1:**
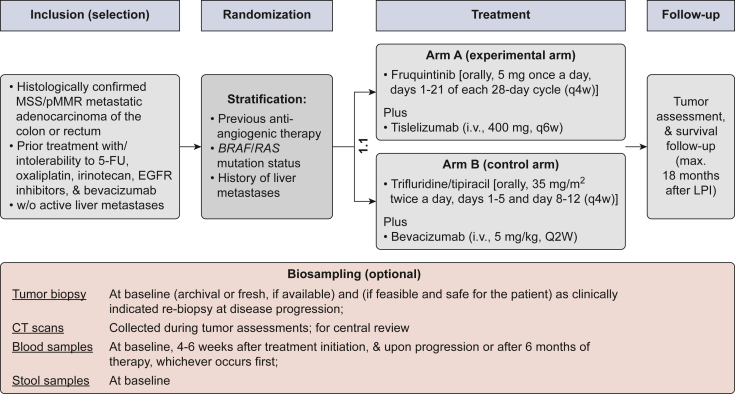
**Study schedule overview.** 5-FU, 5-fluorouracil; CT, computed tomography; EGFR, epidermal growth factor receptor; i.v., intravenous; LPI, last patient in; MSS, microsatellite stable; pMMR, mismatch repair-proficient.

Patients in the experimental arm will receive fruquintinib 5 mg orally once daily on days 1-21 of each 28-day cycle (q4w), in combination with tislelizumab 400 mg intravenously on day 1 of each 42-day cycle (q6w) (Figure 1).

#### Arm B (control arm)

Patients in the control arm will receive trifluridine/tipiracil 35 mg/m^2^ orally twice daily on days 1-5 and 8-12 of each 28-day cycle (q4w), in combination with bevacizumab 5 mg/kg intravenously on day 1 of each 14-day cycle (q2w).

### Assessments

Patients will receive study treatment for up to 15 months. Tumor response will be assessed every 8 weeks (q8w ± 7 days) during treatment and every 12 weeks (q12w ± 14 days) during follow-up until disease progression. Overall, patients will be followed up for a maximum of 18 months after the last patient is enrolled, or until death, withdrawal of consent, or loss to follow-up, whichever occurs first. Patients who provide additional consent for participation in the accompanying translational research program will contribute tumor tissue samples (at baseline and, if feasible, at disease progression), blood samples (at baseline, week 4, and month 6 or at disease progression, whichever occurs first), and stool samples (at baseline). These materials will be used for exploratory translational analyses to identify molecular biomarkers, assess their correlation with clinical outcomes, and explore mechanisms of resistance as outlined below.

### Translational program

An optional translational research program will accompany the trial to identify biomarkers of response and resistance to immunotherapy in mCRC. Archival tumor tissue, longitudinal blood samples, and baseline stool samples will be collected for integrated analyses of circulating tumor DNA (ctDNA), immune phenotypes, microbiota composition, and microbiota-derived metabolite.^18^, ^19^, ^20^, ^21^ Dietary patterns will be assessed longitudinally, and all routine computed tomography scans will be centrally collected for correlative radiologic analyses.^22^

Given the heterogeneity of immunotherapy responses in mCRC, these analyses aim to refine patient stratification beyond clinical features and to elucidate mechanisms underlying treatment response and resistance. The program focuses on three complementary domains: ctDNA dynamics as a molecular biomarker of response,^23^ the diet–microbiota–metabolite axis as a modulator of antitumor immunity,^24^ and systemic and intratumoral T cells as modulators of immunotherapy response in mCRC.^25^

### Efficacy analysis

Primary efficacy will be assessed in the intention-to-treat (ITT) population by comparing PFS between treatment arms using a log-rank test, Kaplan–Meier estimates, and Cox proportional hazards models to derive HRs and 95% CIs. Sensitivity analyses will be conducted in the per-protocol population. The same methodology will be applied to secondary time-to-event endpoints, including OS and duration of response. Binary endpoints (ORR, disease control rate) will be summarized with frequencies and 95% CIs and compared between arms using stratified Cochran–Mantel–Haenszel tests. HRQoL will be analyzed descriptively using the European Organisation for Research and Treatment of Cancer (EORTC) Quality of Life Questionnaire-Core 30 (QLQ-C30) and EuroQol 5-dimension 5-level (EQ-5D-5L) scores. Safety analyses will include all patients receiving at least one dose of study treatment and will summarize adverse events by severity, seriousness, and relationship to study treatment according to Common Terminology Criteria for Adverse Events (CTCAE). Baseline characteristics, treatment exposure, and concomitant medications will be summarized descriptively. Between-group comparisons will use appropriate non-parametric or categorical tests. All statistical tests will be two-sided with a significance level of 5%, without adjustment for multiple testing; exploratory analyses will be descriptive.

The ITT population will be used for primary analyses, the per-protocol population for sensitivity analyses, and the safety population according to treatment received. Data will be reviewed before database lock to finalize analysis sets. Data management will be carried out using an International Council for Harmonisation-Good Clinical Practice (ICH-GCP)-compliant electronic data capture system by the Frankfurt Institute of Clinical Cancer Research, with data archived for at least 25 years in accordance with regulatory requirements.

### Interim analysis

A futility interim analysis for the primary endpoint (PFS) will be carried out after 40 events have occurred, based on a predefined futility boundary indicating a lack of efficacy. The study will continue if the observed HR ≤1.0; if the HR >1.0, the trial will be stopped for futility. Forty events are considered sufficient for this analysis, as the probability of observing HR >1.0 by chance is ∼1.4% assuming a true HR of 0.5, and 5%-15% assuming a true HR between 0.6 and 0.7 (as per sample size assumptions).

### Sample size estimation

This randomized phase II trial aims to estimate the therapeutic efficacy of fruquintinib in combination with tislelizumab in patients with mCRC. The primary endpoint is PFS, defined as the time from randomization to disease progression per RECIST v1.1 or death from any cause. The expected median PFS is 6 months in the control arm and 9 months in the experimental arm, corresponding to an assumed HR of 0.67, which is considered clinically meaningful. Using a one-sided significance level of 10% and 80% power, a total of 111 events are required to detect this difference using a log-rank test. Assuming a 2-year enrolment period, a 1.5-year follow-up period, and an annual dropout rate of 20%, a total of 140 patients (70 per arm) will be randomized. Sample size assumptions are based on historical data.^3^^,^^26^ All statistical computations were carried out using SAS software, Version 9.4 (SAS Institute, Cary, NC).

## Discussion

Considering prior safety and efficacy data from PD-1 + TKI combinations, including the phase III LEAP-017 trial and phase II studies of fruquintinib + tislelizumab, the regimen has shown favorable tolerability and clinically meaningful activity—particularly in non-liver-metastatic CRC. The QUINTIS trial therefore represents a rational next step to confirm these findings in a randomized, controlled setting using an established standard-of-care comparator. The potential clinical benefits are expected to outweigh the manageable safety risks, especially as both agents are already approved and well characterized in this disease context. Patients without a history of liver metastases as well as patients with previously treated liver metastases are included and used for stratification in order to balance any potential negative impact of prior, but treated, liver metastases.

## CRediT author statement

JT is the coordinating investigator for the translational part and contributed to the writing of the protocol and this article. JG recruits patients and contributed to the writing of this article. SEA and TG are representatives of the sponsor. DA, ARS, SK, and GP are members of the steering committee. FD recruits patients and contributed to the writing of this article. MSC and RB contributed to the translational sample collection. AS is the coordinating investigator for the trial and contributed to the writing of the protocol and this article.
