# Supplementary material for: Fruquintinib in combination with tislelizumab versus trifluridine/tipiracil and bevacizumab in third-line and beyond MSS mCRC without active liver metastases—the IKF-080/AIO-QUINTIS trial
Source: ESMO Gastrointest Oncol. 2026 Mar 10;11:100312. doi: 10.1016/j.esmogo.2026.100312 (PMC12994080; doi:10.1016/j.esmogo.2026.100312)
Supplement: Supplementary Table S1 [file mmc1.docx]

**Supplementary Table 1: List of trial sites and PIs**

Germany

Site 49001 Krankenhaus Nordwest, UCT-University Cancer Center, Frankfurt, Germany: Prof. Dr. Thorsten Götze

Site 49003 University Medical Center Hamburg-Eppendorf, II. department of medicine, Hamburg, Germany: Dr. Joseph Tintelnot

Site 49004 Klinikum Mutterhaus der Borromäerinnen, med. clinic I, Trier, Germany : Dr. Ameen Aslan;

Site 49010 LMU Klinikum, Department of Hematology/Oncology, Großhadern, Munich, Germany: Dr. Sabrina Opatz

Site 49015 University of Augsburg, Department of Hematology and Oncology ,Augsburg, Germany: Dr. Frank Jordan

Site 49025 University Hospital Frankfurt, Department of Internal Medicine I, Frankfurt, Germany: PD Dr. Christine Koch

Site 49028 Vincentius-Diakonissen-Kliniken gAG, Karlsruhe, Germany: Alexander Kolov

Site 49029 Klinikum St. Marien Amberg, Amberg, Germany:  Dr. Ludwig Fischer von Weikersthal

Site 49046 Hannover Medical School,, Department of Gastroenterology, Hepatology, Infectious Diseases and Endocrinology, Hannover, Germany: PD Dr. Thomas Wirth

Site 49057 University of Essen, Innere Klinik, Essen, Germany: Prof. Dr. Stefan Kasper

Site 49060 Helios Hospital Bad Saarow, Department of Medical Oncology, Bad Saarow, Germany:PD Dr. Daniel Pink

Site 49065 Klinik Essen-Mitte, Department of Hematology and Oncology, Essen, Germany:: Dr. Christian Müller

Site 49067 Hematology-Oncology Practice Eppendorf (HOPE), Hamburg, Germany : Prof. Dr. Alexander Stein

Site 49069 University of Leipzig, UCCL, Leipzig, Germany: Dr. Benjamin Kobitzsch

Site 49073 MVZ Ravensburg, Ravensburg, Germany: Prof. Dr. Tobias Dechow

Site 49077 TUM University Hospital, Department of Medicine III, Rechts der Isar, Munich, Germany: Prof. Dr. Sylvie Lorenzen

Site 49086 University of Halle (Saale), Innere Medizin I, Halle (Saale), Germany: Dr. Petra Büchner-Steudel

Site 49087 University Hospital Ulm, l. Department of Medicine, Ulm, Germany: Dr. Thomas Ettrich

Site 49155 Leopoldina Krankenhaus, Medizinische Klinik II, Schweinfurt, Germany: Prof. Dr. Stephan Kanzler

Site 49163 Marien Hospital Herne, Germany: PD Dr. Amin Turki

Site 49187 Helios Klinikum Emil von Behring, Department of Hematology and Oncology, Berlin: Dr. med. Börge Arndt

Site 49207 Klinikum Chemnitz, Oncologic Center Chemnitz, Chemnitz, Germany: Jack Chater Cure

Site 49233 University of Düsseldorf, Department of Gastroenterology, Hepatology and Infectious Diseases, Düsseldorf, Germany: Prof. Dr. Christoph Roderburg

Site 49240 Charité Universitätsmedizin Berlin, Medicine Department of Hematology, Oncology and Tumor Immunology, Berlin, Germany: PD Dr. Arndt Stahler

Site 49243 Asklepios Clinic Altona, Department of Hematology, Oncology and Palliative Medicine, Hamburg, Germany: Prof. Dr. Dirk Arnold

Site 49274 St. Josef Hospital Bochum, Department of Hematology and Oncology, Bochum, Germany: Prof. Dr. Anke Reinacher-Schick

Austria

Site 43001 Medical University of Vienna, Division of Oncology, Department of Medicine 1, Vienna, Austria, Prof. Dr. Gerald Prager

Site 43002 Ordensklinikum Linz, Department of med. Oncology and Hematology, Linz, Austria : Dr. Bernhard Doleschal

Site 43005 Paracelsus Medical University, Salzburg, Austria : Prof. Dr. Lukas Weiss

Site 43008 University of Wiener Neustadt, Internal Medicine, Hematology and Oncology, Wiener Neustadt, Austria: Prof. Dr. Birgit Grünberger
